# Prostaglandin E_2_ mediates the late phase of ischemic preconditioning in the heart via its receptor subtype EP_4_

**DOI:** 10.1007/s00380-022-02219-4

**Published:** 2022-12-16

**Authors:** Takayasu Kanno, Naoki Nakagawa, Tatsuya Aonuma, Jun-ichi Kawabe, Koh-ichi Yuhki, Naofumi Takehara, Naoyuki Hasebe, Fumitaka Ushikubi

**Affiliations:** 1 Department of Pharmacology, Asahikawa Medical University, Asahikawa, Japan; 2 Division of Cardiology, Nephrology, Pulmonology and Neurology, Department of Internal Medicine, Asahikawa Medical University, 2-1-1-1 Midorigaoka-Higashi, Asahikawa, Japan; 3 Department of Cardiovascular Regeneration and Innovation, Asahikawa Medical University, Asahikawa, Japan; 4 Division of Integrated Life Science, Department of Biochemistry, Asahikawa Medical University, Asahikawa, Japan

**Keywords:** Ischemic preconditioning, Ischemia–reperfusion injury, Prostaglandin E_2_, EP_4_, Akt pathway

## Abstract

Ischemic preconditioning (IPC) describes a phenomenon wherein brief ischemia of the heart induces a potent cardioprotective mechanism against succeeding ischemic insult. Cyclooxygenase-2 (COX-2), a rate-limiting enzyme in prostanoid biosynthesis, is upregulated in the ischemic heart and contributes to IPC. Prostaglandin E_2_ (PGE_2_) protects the heart from ischemia–reperfusion (I/R) injury via its receptor subtype EP_4_. We sought to clarify the role of the PGE_2_/EP_4_ system in the late phase of IPC. Mice were subjected to four IPC treatment cycles, consisting of 5 min of occlusion of the left anterior descending coronary artery (LAD). We found that COX-2 mRNA was significantly upregulated in wild-type hearts at 6 h after IPC treatment. Cardiac PGE_2_ levels at 24 h after IPC treatment were significantly increased in both wild-type mice and mice lacking EP_4_ (EP_4_^–/–^). At 24 h after IPC treatment, I/R injury was induced by 30 min of LAD occlusion followed by 2 h of reperfusion and the cardiac infarct size was determined. The infarct size was significantly reduced by IPC treatment in wild-type mice; a reduction was not observed in EP_4_^–/–^ mice. AE1-329, an EP_4_ agonist, significantly reduced infarct size and significantly ameliorated deterioration of cardiac function in wild-type mice subjected to I/R without IPC treatment. Furthermore, AE1-329 significantly enhanced the I/R-induced activation of Akt, a pro-survival kinase. We demonstrated that the PGE_2_/EP_4_ system in the heart plays a critical role in the late phase of IPC, partly by augmenting Akt-mediated signaling. These findings clarify the mechanism of IPC and may contribute to the development of therapeutic strategies for ischemic heart disease.

## Introduction

Ischemic preconditioning (IPC) is well documented as a potent cardioprotective phenomenon [1–3]. It refers to a brief ischemic episode of the heart that induces a potent cardioprotective mechanism against succeeding ischemic insult. IPC consists of two phases, an early and a late phase [1–3]. The early phase develops within minutes of an initial ischemic episode and lasts for 2–4 h [1–3]. The late phase begins 12–24 h after the initial ischemic episode and persists for 72–96 h [1–3]. Because the late phase of IPC protects the heart from both myocardial infarction and from stunning for a substantial period of time [4, 5], it has potential clinical relevance. Many previous studies have focused on the complex mechanisms underlying IPC; however, the mechanisms remain to be clarified in detail [6].

The current consensus is that the early phase of IPC is mediated by the activation of a preexisting signaling cascade [7], whereas the late phase results from the synthesis of cardioprotective mediators. Recent studies have elucidated that cyclooxygenase-2 (COX-2), a rate-limiting enzyme for the synthesis of prostanoids, is crucial for the late phase of IPC [8, 9] and atherosclerotic plaque stabilization [10]. Accordingly, the late phase of IPC is abrogated by COX-2-selective inhibitors, such as NS-398 and celecoxib [11]. Upregulation of COX-2 during IPC results in increased synthesis of cardioprotective prostaglandins (PG), such as prostaglandins I_2_ and E_2_ (PGE_2_) [8, 9, 11]. However, it remains to be determined which type(s) of PG participate in the cardioprotection afforded by COX-2 in the late phase of IPC.

PGE_2_ exerts various actions through each of its receptor subtypes (EP_1_, EP_2_, EP_3_, and EP_4_) [12]. It has been reported that EP_4_ mRNA is highly expressed in the hearts of several species, including mouse and human, which suggests that EP_4_ may have some role in the heart. Indeed, studies have shown the upregulation of EP_4_ expression levels in mouse models of myocardial infarction [13]. Importantly, our previous study demonstrated that PGE_2_ exerted a potent cardioprotective effect via EP_4_ in ischemia/reperfusion (I/R) injury [14]. In mice lacking EP_4_ (EP_4_^–/–^), I/R injury was abrogated significantly in an in vivo I/R model and in an ex vivo perfused heart model. This indicated that PGE_2_’s cardioprotective effect was mediated within the heart through the PGE_2_/EP_4_ system.

Furthermore, an EP_4_-specific agonist has been reported to impart a cardioprotective effect by suppressing the expression of macrophage chemoattractant protein-1, thus inhibiting the infiltration of macrophages into the ischemic area [15]. This indicates that EP_4_ in macrophages also can participate in the cardioprotective mechanisms of the PGE_2_/EP_4_ system. While a recent report showed that PGE_2_/EP_4_ activation ameliorates hepatic I/R injury via the ERK1/2/glycogen synthase kinase (GSK) 3β pathway [16], the role of the PGE_2_/EP_4_ system in the IPC remains to be clarified.

We hypothesized that PGE_2_, derived from COX-2, is upregulated by brief ischemic stress and contributes to the late phase of IPC via EP_4_. To test this hypothesis, we examined I/R injury in an EP_4_^–/–^ mouse model of IPC. Using a novel EP_4_-specific agonist, AE1-329, we sought a mechanistic explanation for the cardioprotective function of the PGE_2_/EP_4_ system.

## Materials and methods

### Mice

The details of the breeding and maintenance of animals used in the present study were previously reported [17]. Most EP_4_^–/–^ mice die postnatally as a result of patent ductus arteriosus or do not survive in the C57BL/6 background. Therefore, F2 progenies of surviving EP_4_^–/–^ mice and their wild-type litter mates were independently maintained in a mixed genetic background of 129/Ola and C57BL/6 [18]. All experiments were performed using 7–12-week-old male mice per the guidelines of Japan’s Act on Welfare and Management of Animals and were approved by the Asahikawa Medical University Committee on Animal Research.

### IPC and I/R procedures

The study mice were anesthetized with pentobarbital (60 mg/kg body weight, intraperitoneally) and secured in a supine position with the upper and lower extremities held on a heated table under a two-lead electrocardiogram (ECG) monitoring device. Tracheal intubation with a blunt 20-gauge polyethylene tube (Terumo, Tokyo, Japan) was performed under direct visualization of the tube through the tracheal wall at the upper border of the thyroid cartilage, which was surgically exposed. The tracheal tube was connected to a mechanical ventilator (SN-480-7; Shinano, Tokyo, Japan) and the mice were ventilated using a volume-controlled ventilation mode (0.8 ml room air/breath at 110 breaths/min). After the left anterior thoracotomy, the heart was exposed and the pericardium dissected; the dissection table was tilted up and to the left to visually identify the left coronary artery. An 8–0 nylon suture was passed underneath the left anterior descending coronary artery (LAD) at a position 1 mm from the tip of the left auricle. A small length of polyethylene tube with a blunt edge (size 3; Hibiki, Tokyo, Japan) was threaded by two lines of suture and mounted vertically on the LAD, with a piece of rubber (1 g) attached to each end of the suture. The LAD was occluded by a suture supporting rubber weights and was reopened by manually releasing the weight loading. To confirm that the LAD was successfully occluded, the myocardium was checked for color change (from brick red to pale). In addition, ECG observations showed prolonged QRS duration, enlarged QRS voltage, and ST elevation after successful occlusion [19]. On Day 1, mice in the IPC group underwent the IPC treatment, consisting of four 5-min cycles of occlusion followed by 5 min of reperfusion of the LAD. Mice in the IPC sham group underwent the same surgery, apart from IPC treatment. On Day 2, 24 h after IPC treatment, the mice were subjected to 30 min of LAD occlusion followed by 2 h of reperfusion. To examine the effects of the EP_4_ agonist, AE1-329 [20], I/R injury was induced by occluding the LAD for 30 min, without IPC treatment, followed by indicated times of reperfusion. AE1-329 (30 μg/kg) was injected subcutaneously 30 min before LAD occlusion.

### Reverse transcription polymerase chain reaction analysis for COX-2 mRNA

The hearts of wild-type mice were excised 6 h after IPC or sham treatment and total RNA was prepared from ischemic and nonischemic areas using Isogen (Nippon Gene, Toyama, Japan). Total RNA (2 μg) was reverse-transcribed, as previously reported (10). The resulting cDNA was amplified by polymerase chain reaction (PCR) using primer sets corresponding to COX-2 mRNA, as follows:

sense 5′-ACACTCTATCACTGGCACCC-3′

antisense 5′-GGACGAGGTTTTTCCAC-CAG-3′

The quantity of PCR product was determined by real-time PCR analysis using Lightcycler apparatus (Idaho Technology, Idaho Falls, ID, USA) and DNA Master SYBR Green I (Roche Molecular Biochemicals, Mannheim, Germany) as previously described [20]. The values for the ischemic areas were expressed in relation to the nonischemic areas.

### PGE_2_ enzyme immunoassay

Hearts were excised immediately or 24 h after IPC treatment. Tissue samples were prepared from ischemic (anterior left ventricular wall) and nonischemic (posterior left ventricular wall) areas by homogenization in 0.1 M phosphate buffer containing 1 mM EDTA and 10 μM indomethacin. Prostaglandins were pre-extracted from tissue samples using silica-based octadecylsilane reverse-phase columns. The levels of PGE_2_ in the samples were determined using an enzyme-linked immunoassay kit (Cayman Chemicals, Ann Arbor, MI, USA), according to the manufacturer’s instructions.

### Determination of the area at risk (AAR) and myocardial infarct size

After 30 min of LAD occlusion (with or without IPC treatment) and following reperfusion of 2 h, the size of the AAR and the infarct size were determined using a double-staining technique [21]. Briefly, the right carotid artery was exposed via blunt dissection of the paratracheal muscles, and then cannulated with a catheter. AAR was determined by retrograde injection of 5.0% Evans Blue dye (300 μl) through the catheter while the LAD was occluded. By this procedure, all cardiac tissue except the AAR was stained blue. After the heart was excised and washed in ice-cold phosphate-buffered saline (PBS), it was frozen at − 80 °C for 5 min and cut into five slices. The slices were incubated with 1.0% triphenyltetrazolium chloride (TTC) at 37 °C for 5 min, followed by overnight immersion in PBS at 4 °C. Thus, the infarcted area was demarcated as a pale-to-white area, while viable tissue was stained red. AAR and infarct size were determined via planimetry using Photoshop 7.0 (Adobe, San Jose, CA, USA). The infarct size was calculated as ratio of the infarct (pale-to-white area) to AAR (all cardiac tissue except blue areas) since the perfusion territory of LAD was different in each mouse [21].

### Echocardiographic examination of the cardiac function

Echocardiography was performed before the I/R procedure, and after 2 h of reperfusion following 30 min of LAD occlusion, using a Vevo 660 machine (Primetech; VisualSonics, Toronto, Canada) with a 35-MHz probe. First, a B-mode image of the left ventricle (LV) was obtained in the short-axis view at the level of the papillary muscles. Then, end-diastolic and -systolic LV dimensions were measured from the M-mode tracings. LV ejection fraction (LVEF) was calculated using the equation: LVEF = (LV diastolic volume − LV systolic volume)/LV diastolic volume) × 100, where LV diastolic and systolic volumes indicate left ventricular diastolic and systolic volumes, respectively.

### Western blotting analysis for Akt

Hearts were excised after 15 min of reperfusion following 30 min of LAD occlusion without IPC treatment. The ischemic area was harvested, frozen in liquid nitrogen, and stored at − 80 °C until use. For detection of Ser473-phospholylated Akt (p-Akt) and Akt, the samples were homogenized in lysis buffer (20 mM Tris, 1 mM EDTA, 1 mM DTT, 1% Triton X-100, 2 mM Na_3_VO_4_, 2 mM NaF, 10 mM sodium pyrophosphate, protease inhibitor cocktail; pH 7.5). After centrifugation for 10 min at 12,000 rpm, protein concentrations in the supernatant were determined using the bicinchoninic acid (BCA) Protein Assay Kit (Pierce; Thermo Fisher Scientific, Waltham, MA, USA). The sample (40 μg protein) was electrophoresed using sodium dodecyl-sulfate polyacrylamide gel electrophoresis (SDS-PAGE) and transferred to a polyvinylidene difluoride transfer membrane (Millipore, Billerica, MA, USA) using a semidry transfer system (ATTO, Tokyo, Japan). After a blocking procedure using 5% nonfat dried milk for 1 h at room temperature, the membranes were incubated with the antibodies against p-Akt or Akt (×1000; Cell Signaling Technology) at 4 °C overnight. P-Akt and Akt were detected using horseradish peroxidase-conjugated secondary antibodies (GE Healthcare, Chicago, IL, USA) and an enhanced chemiluminescence reagent. Densitometry of the bands was analyzed using Photoshop as previously described [20].

### Statistical analyses

All data are expressed as mean ± standard error. The data were analyzed using the Student’s *t* test for unpaired samples. *P* values of < 0.05 were considered statistically significant.

## Results

### Augmented COX-2 mRNA expression and PGE_2_ production in the heart after IPC treatment

**Fig. 1:**
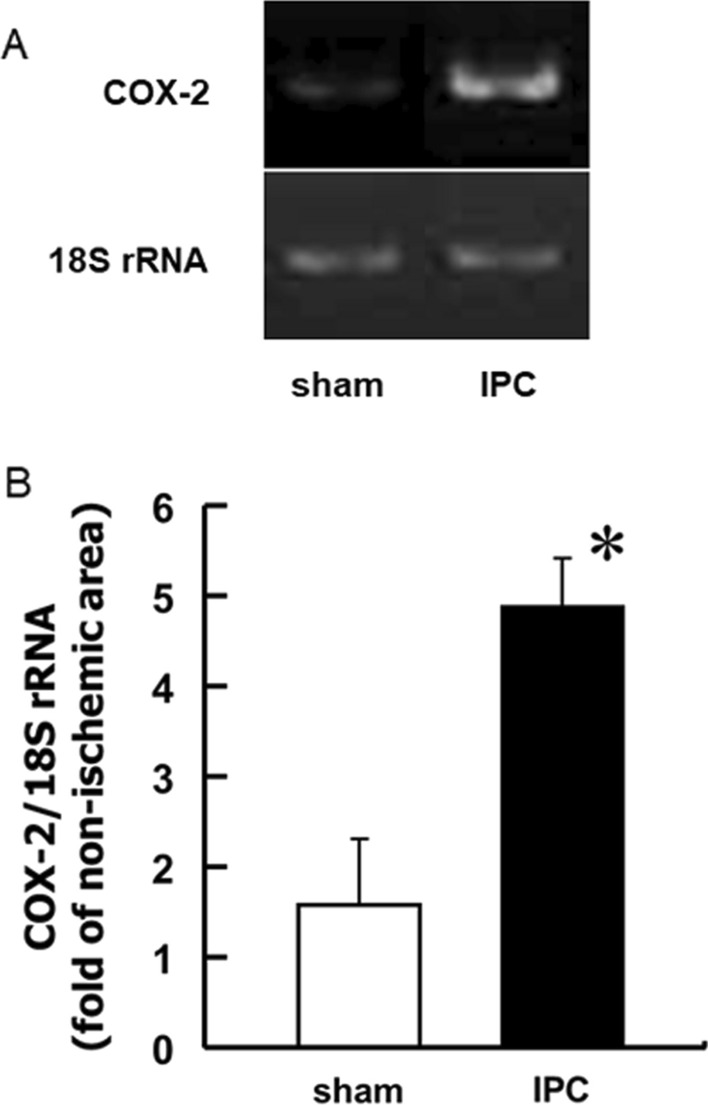
Upregulation of COX-2 mRNA after the IPC treatment. Wild-type hearts received a brief ischemic stress from four 5-min cycles of LAD occlusion, followed by 5 min of reperfusion. At 6 h after IPC treatment, tissue samples were prepared from ischemic and nonischemic areas of the heart, and then examined for the expression of COX-2 mRNA, using **A** RT-PCR or **B** quantitative RT-PCR. The values were expressed as percentages of the nonischemic area, representing the mean values from two independent experiments. *n* = 3. *P < 0.05 vs. sham-operated

**Fig. 2:**
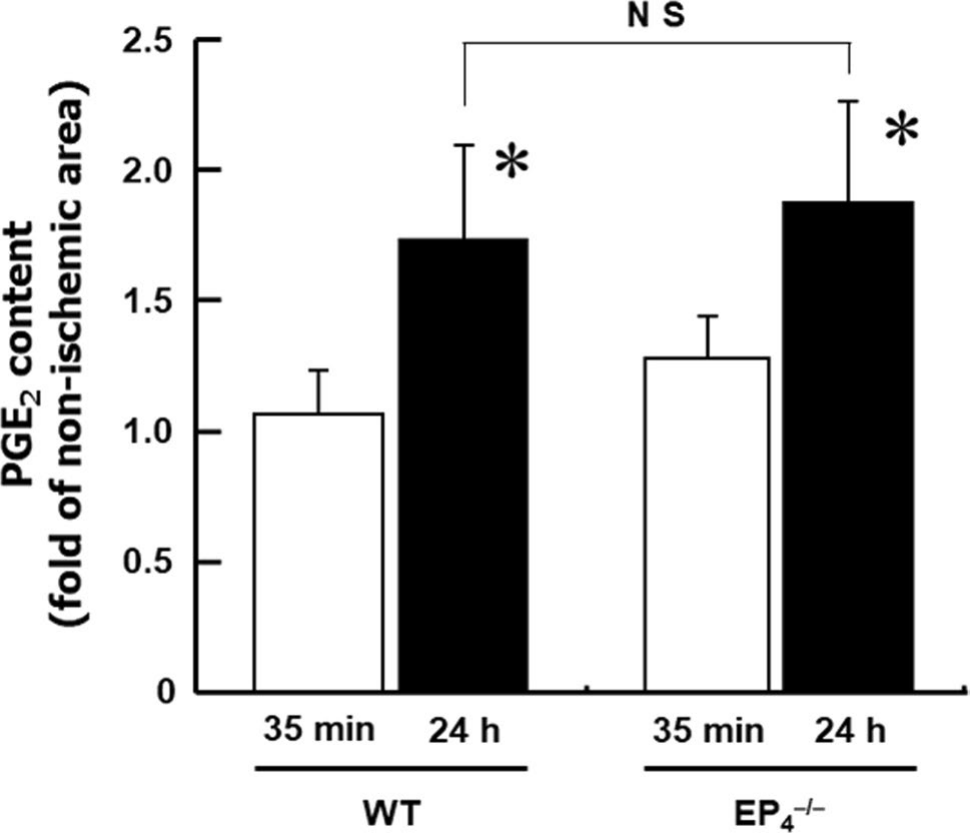
Increased PGE_2_ levels in the heart after IPC treatment. PGE_2_ levels in wild-type and EP_4_^–/–^ hearts were measured immediately and 24 h after IPC treatment. The values were expressed as a percentage of the nonischemic area. *n* = 3–9. *P < 0.05 vs. the non-ischemic area

The expression level of COX-2 mRNA in the heart of sham-operated wild-type mice was low and barely detectable. However, after IPC treatment, the expression level of COX-2 mRNA in the ischemic area increased remarkably at 6 h (Fig. 1), which indicates that the production of cardioprotective prostanoids (such as PGE_2_) increases in the heart subjected to IPC treatment. Immediately after the IPC treatment, PGE_2_ levels in the ischemic area were similar to those of the nonischemic control areas (Fig. 2), which indicated that there was no increase in PGE_2_ production at that time point. However, the PGE_2_ level in the ischemic area increased significantly at 24 h after IPC treatment, compared with that of the nonischemic control area, in both wild-type and EP_4_^–/–^ hearts to a similar degree (Fig. 2), indicating that PGE_2_ may play a role in the late phase of IPC. PGE_2_ levels in the nonischemic areas at 24 h after IPC treatment did not differ significantly between wild-type and EP_4_^–/–^ hearts (5.24 ± 0.73 pg/mg [*n* = 9] and 4.10 ± 0.38 pg/mg [*n* = 6], respectively).

### The late phase of IPC observed in wild-type hearts disappears in EP_4_^–/–^ hearts

**Fig. 3:**
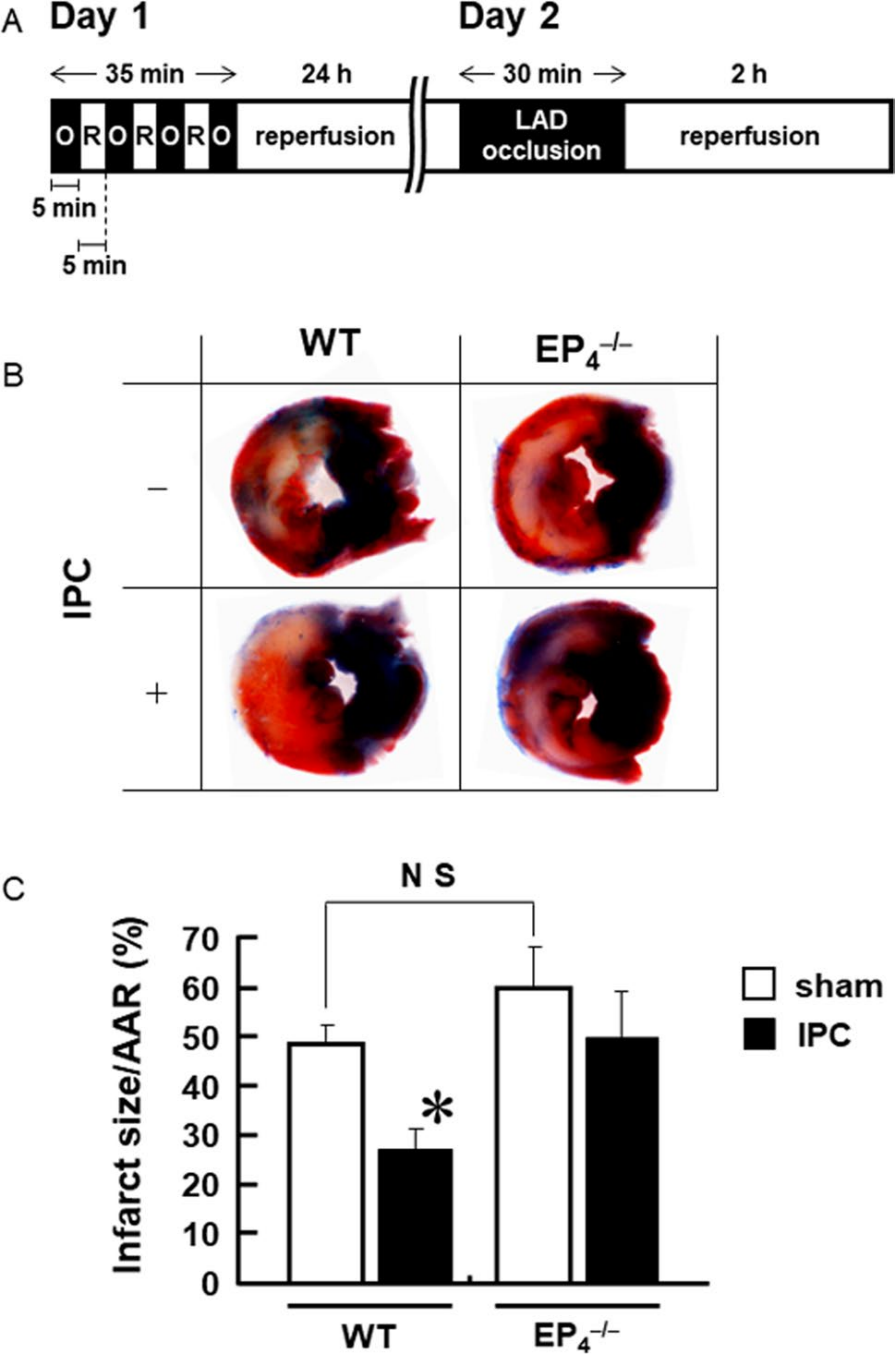
IPC treatment reduces the infarct size in wild-type hearts but not in EP_4_^–/–^ hearts. **A** The experimental protocol for the IPC and I/R procedures (O: LAD occlusion; R: reperfusion). **B** Representative photomicrographs of LV sections from wild-type and EP_4_^–/–^ mice after 2 h of reperfusion following 30 min of LAD occlusion. Tissues that were stained blue (Evans Blue dye) represent nonischemic areas; tissues stained red (TTC) within the ischemic area are live tissues. Unstained tissues appear pale-to-white and represent necrotic myocardium. **C** Cardiac infarct size and AAR were measured in wild-type and EP_4_^–/–^ hearts. The values presenting the infarct size are expressed as percentages of the AAR. *n* = 4–5. *P < 0.01 vs. sham-operated group

To determine the role of PGE_2_ via EP_4_ in late-phase IPC, we performed the I/R procedure after IPC treatment in wild-type and EP_4_^–/–^ mice (Fig. 3A). In wild-type mice, the infarct size of the heart after IPC treatment was significantly smaller than in sham-operated mice (Fig. 3B, C), indicating that the late phase of IPC works effectively in wild-type mice. In contrast, there was no significant difference in the infarct size between the IPC-treated and sham-operated groups in EP_4_^–/–^ mice, indicating that the late phase of IPC disappeared completely in EP_4_^–/–^ mice. In sham-operated groups, there were no significant differences in both infarct size and AAR between wild-type and EP_4_^–/–^ mice (Fig. 3C). These results clearly show that the PGE_2_/EP_4_ system plays a critical role in the late phase of IPC.

### AE1-329, an EP_4_ agonist, reduces the infarct size of wild-type hearts after the I/R procedure without IPC treatment

**Fig. 4:**
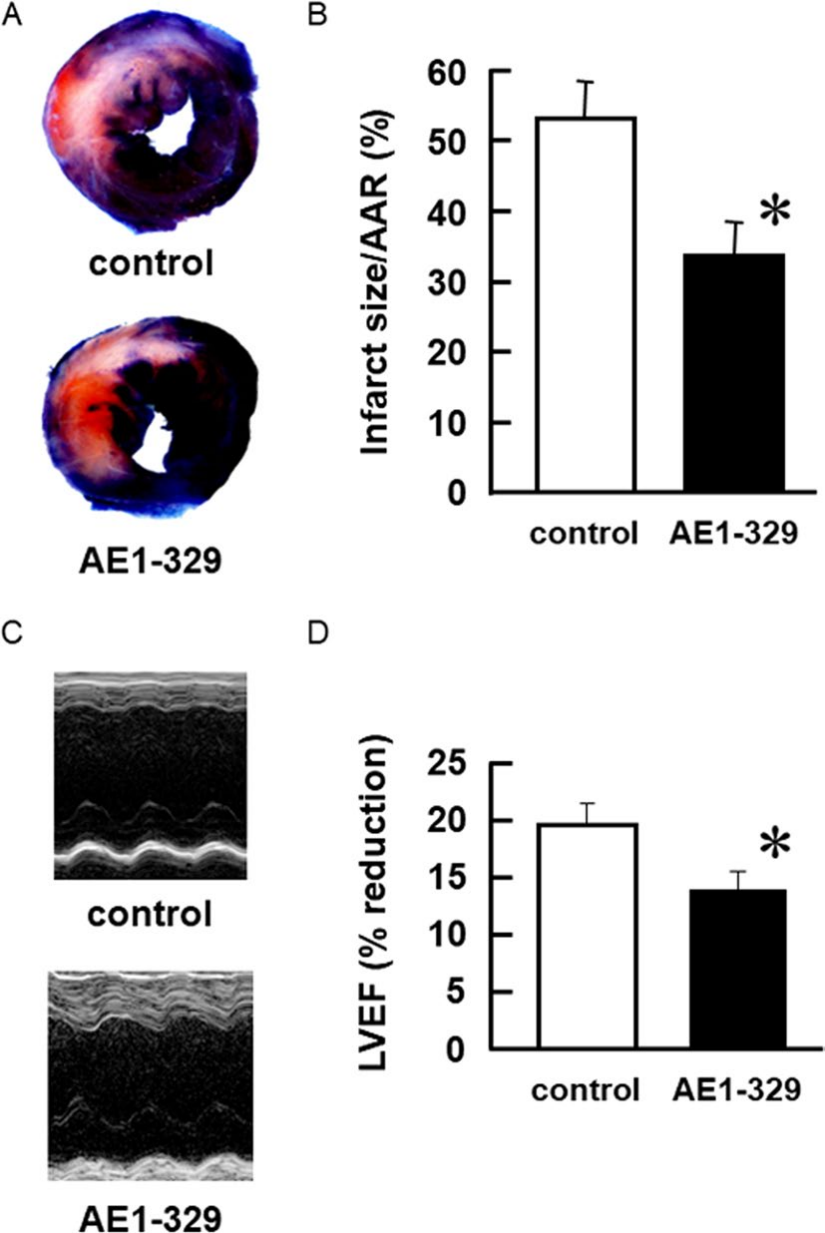
AE1-329, an EP_4_ agonist, reduces infarct size and ameliorates impaired function in wild-type hearts after I/R. **A** Representative photomicrographs of LV sections from control and AE1-329-pretreated mice after 2 h of reperfusion following 30 min of LAD occlusion. **B** Cardiac infarct size and AAR were measured. The values presenting the infarct size are expressed as percentages of the AAR. *n* = 6. *P < 0.01 vs. control. **C** Representative M-mode tracings of the LV from control and AE1-329-pretreated mice after 2 h of reperfusion following 30 min of LAD occlusion. **D** LVEF was measured. *n* = 5–6. *P < 0.05 vs. control

To clarify whether insufficient production of PGE_2_ in the heart was responsible for the lack of significant difference in infarct sizes between sham-operated wild-type and EP_4_^–/–^ hearts, we used AE1-329 to activate EP_4_ in wild-type hearts that did not receive IPC treatment. AE1-329 (30 mg/kg) was injected 30 min before the 30-min LAD occlusion and the infarct size was determined after 2 h of reperfusion. AE1-329 significantly reduced the infarct size in wild-type mice (Fig. 4), whereas no such effect was observed in EP_4_^–/–^ mice (data not shown). In contrast to the efficient activation of EP_4_ and the resultant reduction in the infarct size in the heart with the IPC treatment (Fig. 3C), this result indicates that the endogenous production of PGE_2_ in hearts without IPC treatment was insufficient to activate EP_4_, at least during the 2 h of the I/R procedure. AE1-329 did not significantly affect blood pressure (data not shown).

We used echocardiography to examine the effects of AE1-329 pretreatment on the function of wild-type hearts at 2 h of reperfusion following 30 min of LAD occlusion. In the hearts pretreated with AE1-329, movement of the anterior wall was well retained, compared with that of control hearts (Fig. 4C). Additionally, AE1-329 significantly prevented a reduction in the LVEF after the I/R procedure (Fig. 4D), which indicated that the activation of EP_4_ protected the heart from I/R injury, both histologically and functionally.

### AE1-329 enhances I/R-induced activation of Akt

**Fig. 5:**
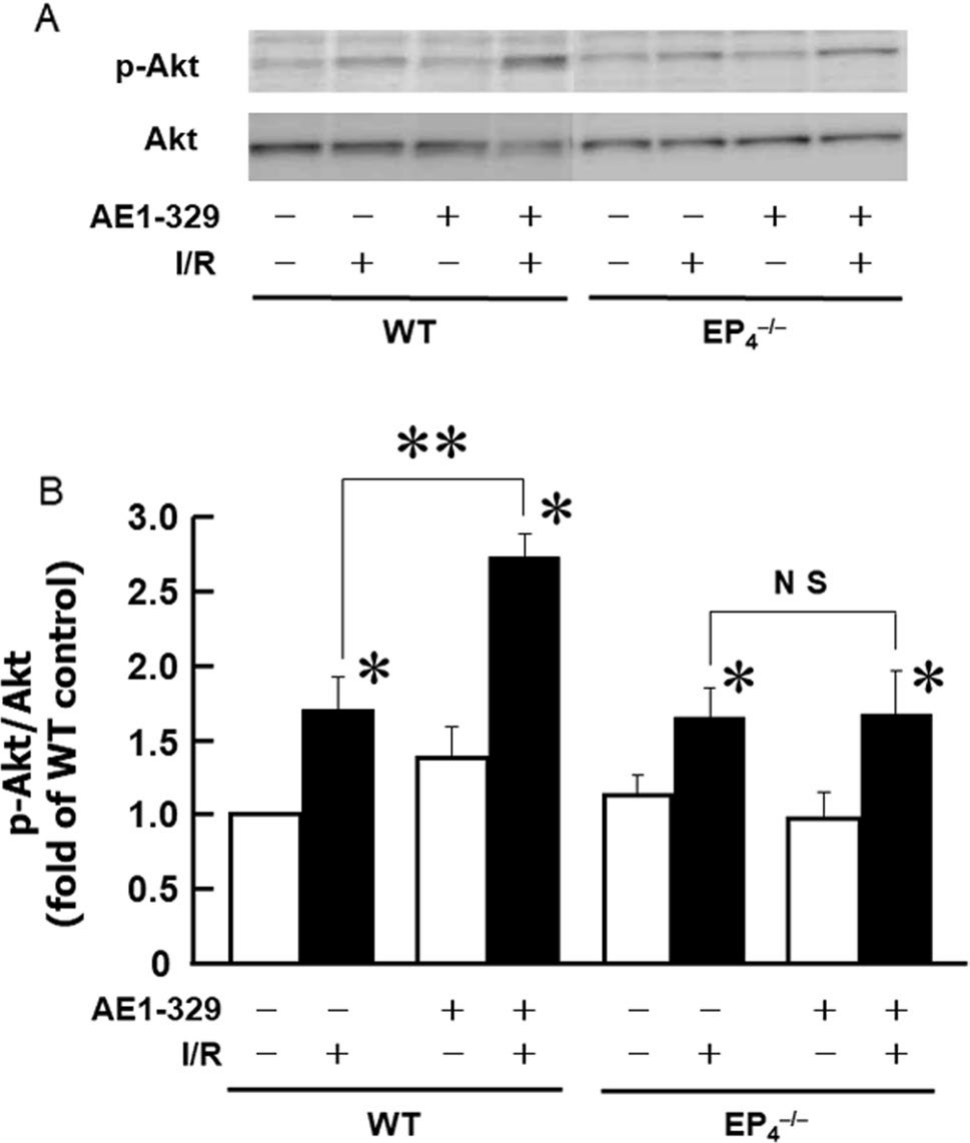
AE1-329 enhances the I/R-induced activation of Akt. Akt and p-Akt levels in wild-type and EP_4_^–/–^ hearts were determined at 15 min of reperfusion following 30 min of LAD occlusion. **A** A representative result of Western blotting showing the levels of Akt and p-Akt. **B** Effects of AE1-329 on the activation of Akt after I/R. Molecular weights of both p-Akt and Akt are 60 kD. The ratio of p-Akt to Akt is expressed as a percentage of that of the nonischemic wild-type control heart. *n* = 3–5. *P < 0.05 vs. respective AE1-329-untreated control. **P < 0.01

To further investigate the cardioprotective mechanism of the PGE_2_/EP_4_ system, we examined the activation of Akt, a pro-survival serine/threonine kinase. We measured the levels of phosphorylated Akt (p-Akt), an activated form of Akt. Although AE1-329 alone did not alter the level of p-Akt, it significantly enhanced the I/R-induced increase in p-Akt level in wild-type hearts (Fig. 5), the enhancement of Akt activation by AE1-329 was not observed in EP_4_^–/–^ hearts (Fig. 5). Both the molecular weight of p-Akt and Akt is 60 kD, suggesting that the upper bands in p-Akt were non-specific. The levels of total Akt were not different between wild-type and EP_4_^–/–^ hearts, irrespective of the presence or absence of AE1-329. These results suggested that Akt signaling underlies the cardioprotective mechanism of the PGE_2_/EP_4_ system in I/R. Meanwhile, I/R treatment increased p-Akt/Akt ratio in EP4^−/−^ mice, suggesting that Akt activation might be increased via pathways other than PGE_2_/EP_4_ system.

## Discussion

Our results show that the PGE_2_/EP_4_ system plays a critical role in the heart in the late phase of IPC, partly by augmenting Akt-mediated signaling. IPC treatment significantly increased the cardiac expression level of COX-2 mRNA and the production of PGE_2_, thus inducing a potent late phase of IPC in wild-type hearts. However, in EP_4_^–/–^ hearts, the late phase of IPC did not establish, which indicates that the PGE_2_/EP_4_ system critically mediates the late phase of IPC. We have previously reported that PGE_2_ protects the heart from I/R injury via EP_4_. We found that the infarct size was significantly larger in EP_4_ hearts than that in wild-type hearts at 24 h of reperfusion following 1 h of LAD occlusion. However, the present study found no significant difference in the infarct size between sham-operated wild-type and EP_4_ mice at 2 h of reperfusion following 30 min of LAD occlusion. This suggests that PGE_2_ production in the heart, without the IPC treatment, was insufficient to activate EP_4_ at this time point; effective activation of EP_4_ and a resultant decrease in the infarct size were observed in wild-type hearts with IPC treatment. Activation of EP_4_ by exogenous AE1-329 significantly reduced the infarct size and rescued the functional deterioration induced by I/R at the same time point. Taken together, these results support a hypothesis that PGE_2_ originating from COX-2, upregulated by a brief ischemic stress, contributed critically, via EP_4_, to the late phase of IPC. Several reports have demonstrated that EP_4_ signaling provides protection from myocardial I/R injury [14, 15, 22]. This is the first study to demonstrate that COX2 upregulation by IPC attenuated ischemic injury through PGE_2_/EP_4_ signaling. Further studies are warranted to clarify the role of PGE_2_/EP_4_ signaling in the late phase of IPC.

Although a cardioprotective role of the PGE_2_/EP_4_ system in I/R injury has been reported previously [14], its precise mechanism remained to be clarified. During myocardial I/R injury, EP_2_ and EP_4_ play a cardioprotective role after ischemia through the activation of the cyclic AMP/protein kinase A signaling pathway [23]. It is known that phosphatidylinositol 3-kinase (PI3-K)/Akt signaling plays an important antiapoptotic and cardioprotective role in cardiac I/R injury [6, 24–26]. Indeed several agents capable of protecting the heart from I/R injury activate PI3-K/Akt signaling when given at reperfusion, such as insulin [27], erythropoietin [28], and bradykinin [29]. A recent study demonstrated that miR-486-5p, a cardioprotective microRNA that can activate the phosphotidylinositol 3-kinase/Akt signaling pathway, was dysregulated in rat models of acute myocardial infarction and in patients with acute myocardial infarction [30].

It has been reported that PGE_2_ activates Akt via EP_4_ in several types of cells, such as human embryonic kidney cells expressing EP_4_ [31], glomerular epithelial cells [32], and human lung carcinoma cells [33]. This suggests the possibility that the PGE_2_/EP_4_ system is also able to activate Akt during cardiac I/R, thus protecting the heart. In the present study, AE1-329 significantly augmented the I/R-induced activation of Akt in wild-type hearts, while no such effect was observed in EP_4_^–/–^ hearts. This suggests that Akt signaling underlies the cardioprotective mechanism of the PGE_2_/EP_4_ system in I/R. Additionally, several studies demonstrated the utility of EP_4_ as a promising therapeutic target in cardiac diseases including not only ischemic heart disease but also inflammatory heart disease [34] and cardiac hypertrophy [35]. Further investigations are necessary to extend the clinical benefits of EP_4_ agonists in cardiac disease.

This study has several limitations. We did not evaluate cellular necrosis and apoptosis although the signals downstream of the phosphotidylinositol 3-kinase/Akt pathway confers necrosis and apoptosis. Further studies should be considered to clarify the molecular mechanism underlying the cardioprotective role of the EP_4_/Akt pathway.

In conclusion, we demonstrated that the PGE_2_/EP_4_ system in the heart plays a critical role in the late phase of IPC, partly by augmenting Akt-mediated signaling. Our findings clarify the mechanism of IPC and could contribute to the development of therapeutic strategies for ischemic heart disease.
